# ﻿Taxonomy and nomenclature of *Abutilonalbidum* (Malvaceae, Malvoideae), a cryptic Saharo-Canarian species recently rediscovered in Tenerife

**DOI:** 10.3897/phytokeys.221.95907

**Published:** 2023-03-07

**Authors:** Filip Verloove, Alexander N. Sennikov, J. Alfredo Reyes-Betancort

**Affiliations:** 1 Meise Botanic Garden, Nieuwelaan 38, B-1860, Meise, Belgium Meise Botanic Garden Meise Belgium; 2 Botanical Museum, Finnish Museum of Natural History, University of Helsinki, P.O. Box 7, 00014, Helsinki, Finland University of Helsinki Helsinki Finland; 3 Jardín de Aclimatación de La Orotava, Instituto Canario de Investigaciones Agrarias (ICIA), C/ Retama 2, 38400, Puerto de la Cruz. Santa Cruz de Tenerife, Tenerife, Spain Instituto Canario de Investigaciones Agrarias Tenerife Spain

**Keywords:** *Abutilon*, Canary Islands, Malvaceae, nomenclature, Saharo-Canarian, taxonomy, Tenerife

## Abstract

*Abutilonalbidum*, a cryptic Saharo-Canarian species, was thought to have been last collected in 1945 in Tenerife by E.R. Sventenius. In 2019, it was rediscovered in the same area. The characteristic features of the Canarian plants are discussed, especially in relation to the morphologically similar-looking and probably closely-related species *Abutilonindicum* and *A.bidentatum*. It is concluded that the plants from Tenerife and north-western Africa indeed represent a distinct species. The species is illustrated and a key for the identification of this and related species is presented.

## ﻿Introduction

*Abutilon* Mill. (Malvaceae, Malvoideae) is a large genus which mostly occurs in tropical and subtropical areas around the world, with a few species extending to warm-temperate regions. The genus is most diverse in the Neotropics (Areces Berazaín and Fryxell 2007). It is one of the largest Malveae genera, although the number of its recognised species considerably varies, depending on the species delimitation. Most authors nowadays accept ca. 150–160 species (e.g. Bayer and Kubitzki (2003); Mabberley (2008); Verdcourt and Mwachala (2009)), but the boundaries of the genus remain unclear.

*Abutilon* lacks a solid, modern revisionary worldwide treatment, as well as an extended molecular phylogeny (a partial molecular phylogeny was presented by Donnell et al. (2012)). However, local accounts are available that cover large parts of the main areas of occurrence of the genus. For the New World, keys were published for the North and South American species (Kearney 1955, 1958; Fryxell and Hill 2015), as well as for the Mexican species (Fryxell 1988). Several accounts are available for the Caribbean as well (e.g. Adams (1972); Fryxell (1989); Areces Berazaín and Fryxell (2007)). In the Old World, taxonomic information is more fragmented and revisions covering multiple countries are scarce. In Africa, useful treatments are available for East Africa (Vollesen 1995; Thulin 1999; Verdcourt and Mwachala 2009), West Africa (Hutchinson and Dalziel 1958), southern Africa (Meeuse 1961; Roessler 1969; Exell and Gonçalves 1979), Central Africa (Hauman 1963) and North Africa (several accounts, for example, Quézel and Santa (1963); Ali and Jafri (1977); Fennane et al. (1999); Boulos (2000); El Hadidi (2000)). In Asia, the revisions for Malesia (Borssum Waalkes 1966), Pakistan (Abedin 1979), China (Tang et al. 2007), Oman (Ghazanfar 2003), Iraq (Townsend 1980), Iran (Riedl 1976), Yemen (Wood 1997) and India (Naqshi et al. 1988; Paul and Nayar 1988; Paul 1993) are worth mentioning. Some useful accounts were also published for islands in the Indian Ocean (e.g. Hochreutiner (1955), Marais (1987)). Finally, in Australia revisions are available for some of the States, such as New South Wales (Mitchell and Norris 1990). In Europe, not a single species is native, although *A.theophrasti* Medik. is sometimes mistakenly considered as such (e.g. Webb (1968)).

In their monumental “Histoire Naturelle des îles Canaries”, Webb and Berthelot (1836) provided a detailed description of “*Abutilonalbidum* Nob.”, a species they explicitly based on Willdenow’s *Sidaalbida* Willd. It was reported from the Santa Cruz area in the south-eastern corner of the island of Tenerife. Subsequent authors (e.g. Bornmüller (1904); Pitard and Proust (1908); Lindinger (1926); Burchard (1929)) regularly confirmed its presence in a small area roughly located between Santa Cruz and Igueste de San Andrés. However, after the 1940s, it was no longer collected nor mentioned in the regional literature. Surprisingly and apparently without any explanation, Canarian records of this species were later considered to be referrable to the invasive weed *A.grandifolium* G. Don (e.g. Eriksson et al. (1974); Santos (1983); Hohenester and Welss (1993); Valdés (2011); Dobignard and Chatelain (2012); BIOTA (2023)).

In December 2019, an unknown species of *Abutilon* was observed by one of us (F.V.) in several localities in the village of Igueste de San Andrés (Municipality of Santa Cruz de Tenerife), on the verge of the natural protected area ‘Anaga Rural Park’ in the south-easternmost part of Tenerife. The observed plants clearly differed from another species of *Abutilon* that is widely naturalised and invasive in the Canary Islands, *A.grandifolium*. Although identification attempts were at first unsatisfactory, the plants eventually were determined to belong to the species that had been known from Tenerife since the end of the 18^th^ century, but which had not been collected since 1945 and apparently completely forgotten by Canarian authors.

## ﻿Materials and methods

Fieldwork was undertaken by the first author in December 2019 and March 2022 and, for revision of other localities in Tenerife, by the second author in May and June 2021. Herbarium specimens were collected and deposited at the herbarium of the Meise Botanic Garden [**BR**; for herbarium acronyms Thiers (2023) is followed]. In addition, numerous photographs were made that showed essential diagnostic features, which are less easily observed in pressed specimens. In March 2022, a detailed inventory was made of all remaining populations and all were georeferenced and registered in the nature observations platform Observation.org (https://observation.org/soort/view/1029740).

Herbarium specimens relevant for this study (including type material) were studied in the herbaria of the Meise Botanic Garden (**BR**), the Jardín Botánico Canario Viera y Clavijo (**LPA**) and the Jardín de Aclimatación de la Orotava (Instituto Canario de Investigaciones Agrarias) (**ORT**). In addition, images from several herbaria available online were also consulted (**B**, **COI**, **FI**, **G**, **K**, **L**, **MO**, **MPU**, **NYBG**, **P**, **RAB**, **UM** and **W**).

Countless literature sources (including protologues), deemed useful for this study, were checked to better understand the identity and taxonomy of the species and the characters that differentiate it from closely-related taxa.

## ﻿Results

### ﻿The nomenclature of *Abutilonalbidum*

Willdenow (1809) validly published the name *Sidaalbida* Willd. for a species that was later transferred to *Abutilon* by Sweet (1826) as *A.albidum* (Willd.) Sweet. The only original extant specimen for *S.albida* is seemingly the holotype (Fryxell 2002) and is preserved at B. From this specimen and the protologue, it can be deduced that the plant described as *S.albida* definitely differs from the Saharo-Canarian one.

However, since there is no other binomial for the Saharo-Canarian plant, conservation of the name *Sidaalbida* with a conserved type was proposed (Verloove and Sennikov (2022) and discussion therein). If the proposal is not accepted, a new name will have to be given to this species.

### ﻿The quest for the identity of an unknown *Abutilon* from Tenerife

From Tenerife, only a single, yellow-flowered species of *Abutilon* is currently known, *A.grandifolium*. This species, native to South America, is widely naturalised and invasive in the Canary Islands. Plants found in 2019 in the south-eastern part of Tenerife are strikingly different. In comparison to *A.grandifolium*, their corollas are much smaller, fruit (schizocarp) has more numerous mericarps (usually around 15), calyx is much shorter than the fruit, stem indumentum is quite different (a mixture of short stellate hairs and long patent hairs) and inflorescences consist of both solitary flowers and axillary (pseudo-)panicles. Yet, since the second half of the 20^th^ century, Canarian authors considered historical records of *A.albidum* to be erroneous and referred them to *A.grandifolium* instead (Eriksson et al. 1974; Santos 1983; Hohenester and Welss 1993; Valdés 2011; Dobignard and Chatelain 2012; BIOTA 2023).

If the Tenerife plant is not *Abutilongrandifolium*, then what is its correct name? Identification attempts initially led – with not too much difficulty – to *A.indicum*, a common weed from the Old World tropics (e.g. Borssum Waalkes (1966)) that is also widely naturalised in the Caribbean (Fryxell 1989). However, the Tenerife plant clearly differed from that species as well, for instance, in having much shorter petals and mericarps. Its stem indumentum is also quite different: it is composed of a mixture of very short, dense stellate hairs, sparser and slightly longer multicellular hairs and rather numerous long, patent simple hairs, whereas in *A.indicum* the stem is densely covered with stellate hairs, rarely with some additional slender, simple hairs.

Identification keys from Pakistan and India key out *Abutilonindicum* together with a similar species, *A.bidentatum* (Hochst.) A. Rich., described from Ethiopia, but widely distributed from Central Africa to India. In areas where these two species occur sympatrically, they are considered to be very similar. Husain and Baquar (1974) separated *A.bidentatum* from *A.indicum*, based on its smaller, paler corollas. Bhandari (1995) distinguished *A.bidentatum* and *A.indicum*, based on carpel length (6–8 mm vs. 9–12 mm, respectively). According to Paul and Nayar (1988), both species are differentiated as follows: *A.bidentatum* has a staminal column 2–3 mm long, a schizocarp 10 mm across, mericarps 10 × 5 mm and gradually acuminate dorsally, whereas *A.indicum* has a staminal column 5–7 mm long, a schizocarp 1.5–2.5 cm across, mericarps 10–15 × 7–10 mm and acute to acuminate dorsally. The diagnostic characters put forward by these authors for *A.bidentatum* are evidently more in line with those observed in the plants found in Tenerife. However, while *A.bidentatum* has mericarps with two distinct dorsal cusps (hence the species epithet), the mericarps in the Tenerife plants are merely shortly acute-triangular dorsally at maturity, without clear protuberances.

Eventually, floristic accounts from north-western Africa, more precisely from Algeria and Morocco (Quézel and Santa 1963; Fennane et al. 1999), which are geographically close to Tenerife, threw new light on the possible identity of the Tenerife plant. Based on mericarp number and stem indumentum, the plants were easily keyed out as Abutilonalbidumsubsp.albidum. A subsequent study of herbarium specimens (including type material) and protologues of *Sidaalbida* (Willdenow 1809), *S.bidentata* (Hochstetter 1842) and *S.indica* (Linnaeus 1756) demonstrated that these three species are very similar in many characters. Yet, the plant material from Tenerife and north-western Africa is considered to be sufficiently distinct, as correctly assumed by Webb and Berthelot (1836) and other Canarian workers up to the first half of the 20^th^ century (e.g. Bornmüller (1904); Pitard and Proust (1908); Lindinger (1926); Burchard (1929)) and by contemporary North African authors (Fennane et al. 1999). Preliminary results from a molecular analysis also showed these three species to be closely related, but distinct (Verloove, unpubl. data).

Thus, *Abutilonalbidum*, *A.bidentatum* and *A.indicum* are very similar in general appearance and have often been confused. Claims of the last from Africa (e.g. Masters (1868); Ulbrich (1913)), for instance, are probably erroneous (e.g. Hauman (1963); Roessler (1969); Verdcourt and Mwachala (2009); Valdés (2011)). Most records belong, in fact, to *A.mauritianum* (Jacq.) Medik. and related or similar species like *A.bidentatum*. *A.albidum* has also been combined under *A.indicum*, as var. albidum (Willd.) Baker f. (Baker 1893).

### ﻿Diagnostic characters

Morphological features used for the separation of *Abutilonalbidum*, *A.bidentatum* and *A.indicum* are discussed below.

The **stem indumentum** of these species is different. *Abutilonindicum* has an indumentum that almost entirely or even exclusively consists of very short and dense stellate hairs. Simple, long patent hairs are always absent or very sparse (e.g. Hochreutiner (1902); Adams (1972); Townsend (1980); Fryxell (1989)). *Abutilonalbidum* and *A.bidentatum*, on the contrary, have a mixture of short stellate hairs and long patent hairs; both hair types are equally abundant. In addition, rather numerous multicellular hairs are observed (some of them gland-tipped). In length, these hairs are slightly or much longer than the stellate ones. This feature is rarely mentioned in floristic accounts, although exactly the same pubescence is observable in the syntype of *A.bidentatum* kept at BR. Notably, Hochreutiner annotated a syntype at K in 1899: “Le A.bidentatum de Hochst. est une forme villeuse et légèrement glanduleuse de *A.indicum* Sw. avec lequel nous l’assimilons” [translation F.V.: *A.bidentatum* of Hochst. is a villous and slightly glandular form of *A.indicum* Sw. with which we equate it.]. Apparently, Hochreutiner also noticed the presence of the third hair type in *A.bidentatum*. Wood (1997) described *A.bidentatum* as a glandular perennial and this likely also refers to the presence of multicellular hairs. The long description of Webb and Berthelot (1836) did not refer to the presence of multicellular hairs. However, such hairs are observable in historical, as well as recent, collections from Tenerife.

These three species also differ in **flower size** and **colour**. The populations found in Tenerife are small-flowered: flowers are ca. 15 mm in diameter with petals 7–10 mm long (i.e. only slightly longer than the calyx). *Abutilonindicum*, in contrast, is a large-flowered species with corollas 25–35 cm in diameter and with petals 12–15 mm long (e.g. Borssum Waalkes (1966); Abedin (1979); Naqshi et al. (1988); Philcox (1997); Areces Berazaín and Fryxell (2007); Bano and Deora (2018)). Petals are, in fact, about twice as long as the calyx or even longer (Riedl 1976). In the syntype of *A.bidentatum* at BR, flowers are about 18 mm in diameter. In addition, the staminal column is much shorter in *A.albidum* and *A.bidentatum* than in *A.indicum* (e.g. Abedin (1979); Naqshi et al. (1988); Paul and Nayar (1988)). The plants from Tenerife and a syntype of *A.bidentatum* at BR have a staminal column 2–3 mm long, whereas the staminal column in *A.indicum* is 5–7 mm long. Finally, petals of *A.albidum* and *A.bidentatum* tend to be slightly paler; i.e. pale yellow to yellow (‘pallidè luteola’ for *A.albidum*, according to Webb and Berthelot (1836)), whereas those of *A.indicum* are usually said to be yellow to orange yellow (e.g. Husain and Baquar (1974); Abedin (1979); Naqshi et al. (1988)).

**Fruit** characters are also different in these species. Mericarps are invariably longer and wider in *Abutilonindicum*. In areas where this species and *A.bidentatum* occur sympatrically they are distinguished (often even exclusively) based on this character (e.g. Jafri (1966); Paul and Nayar (1988); Bhandari (1995)). In the plants from Tenerife, mericarps are up to 9 mm long and 5 mm wide (often slightly smaller). This is roughly in line with measurements taken on the syntype of *A.bidentatum* at BR (8 mm long and 5 mm wide). In *A.indicum*, in contrast, mericarps are often twice as long and broader. The number of mericarps per fruit tends to be higher in *A.indicum* than in *A.albidum* and *A.bidentatum*. In *A.albidum*, the number of mericarps ranges between 10 and 15 (Webb and Berthelot 1836), although most fruits have around 15 mericarps. Similar numbers are known for *A.bidentatum*, whereas Philcox (1997) gives 20–30 for *A.indicum*. We observed some overlap in these measurements, but the mericarps are, indeed, more numerous in *A.indicum*. In all these species, mericarps turn black at maturity; they are hairy outside and shiny inside. Mericarp ornamentation is considered to be an important diagnostic feature for the separation of species in *Abutilon*. The outer apical (dorsal) margin can be either rounded (without protuberances) or angled. In the latter case, the outgrowth can be merely acute, gradually acuminate to aristate. *Abutilonbidentatum* was initially described as having mericarps that are bidentate at apex (“carpellis compressis apice truncatis, bidentatis”). In the syntype at BR, a cusp ca. 1 mm long is discernible. Most authors describe the cusp length as 1–2 mm long (e.g. Thulin (1999); Verdcourt and Mwachala (2009)). At this point, the plants observed in Tenerife definitely differ from *A.bidentatum*: mericarps are angled at the dorsal margins, but a cusp or protuberance is missing. In *A.indicum*, mericarps can vary from rounded, obtuse to long acuminate (Borssum Waalkes (1966); see also Bano and Deora (2018)). The schizocarp also tends to be less wide in *A.albidum* and *A.bidentatum* (10–15 mm across) than in *A.indicum* (rather 15–25 mm across). Finally, according to Jafri (1966), mericarps in *A.bidentatum* already dehisce before breaking away from the central axis – a feature also observed in the plants from Tenerife – whereas in *A.indicum* mericarps dehisce after breaking away from the central axis.

Finally, *Abutilonalbidum*, *A.bidentatum* and *A.indicum* also differ in **inflorescence shape**. All have flowers that are inserted in the leaf axils. However, whereas in *A.indicum* flowers are invariably solitary, they often merge into distinct panicles in *A.bidentatum* (e.g. Verdcourt and Mwachala (2009)). This feature was also observed in the plants that are found in Tenerife.

On the other hand, some diagnostic characteristics mentioned in literature proved to be of no value or merely erroneous. For instance, pedicels are usually said to be shorter than petioles in *A.bidentatum* and vice versa in *A.indicum* (Abedin 1979; Borssum Waalkes 1966; Ghazanfar 2003; Verdcourt and Mwachala 2009). In reality, pedicel and petiole length are variable in these three species, although pedicels often are at least as long as or longer than petioles, also in *A.bidentatum* (as can be seen in a syntype at BR, as well as in the material from Tenerife).

The features that appear to be most reliable for the separation of these three species are summarised in Table 1.

**Table 1.** Features considered most reliable for differentiating *Abutilonalbidum*, *A.bidentatum* and *A.indicum* (based on our study of type material, Borssum Waalkes (1966); Abedin (1979); Vollesen (1995); Ghazanfar (2003); Verdcourt and Mwachala (2009); Randall (2017) and many other sources).

Character / species	*Abutilonalbidum*	*A.bidentatum*	*A.indicum*
Stem pubescence	Stellate, tomentose, intermixed with long simple patent hairs and multicellular hairs	Stellate, tomentose, intermixed with long simple patent hairs and multicellular hairs	Dense stellate hairs, rarely with some slender, simple hairs
Staminal column length	2–3 mm long	2–3 mm long	5–7 mm long
Mericarps	10–15 in number, 9 × 5 mm, the outer apical (dorsal) margin acute-triangular, without cusps or protuberances	13–16 in number, 8–10 × 3–5 mm, the outer apical (dorsal) margin bidentate, i.e. with two cusps 1–2 mm long	15–22 in number, 10–18 × 7–9 mm, the outer apical (dorsal) margin either rounded, acute or long-acuminate
Corolla	10–15 mm across, petals ca. 8 mm long, pale yellow to yellow	10–15 mm across, petals ca. 8 mm long, pale yellow to yellow	25–35 mm across, petals 12–15 mm long, yellow to orange yellow
Inflorescence	Flowers solitary in the leaf axils or merging into distinct panicles	Flowers solitary in the leaf axils or merging into distinct panicles	Flowers solitary, in the leaf axils
Distribution	Canary Islands (Tenerife), north-western Africa	Native to eastern Africa	Native to the Indian Subcontinent and neighbouring territories; introduced elsewhere
Ecology	Basaltic rocks in the desert or semi-desert, sandy river beds	Riverine forest, river banks, alluvial Acacia wooded grassland and bushland; weedy in Egypt, Saudi Arabia and India	Waste places, roadsides, along the beach, as a weed in plantations and gardens

From the above, it can be concluded that *Abutilonalbidum* is most closely similar to *A.bidentatum* morphologically. These two species share all characters, except that their mericarps are ornamented in different ways. Since mericarp ornamentation is considered to be an important diagnostic trait in the genus (Alzahrani et al. 2021), both species are apparently distinct. Besides these morphological differences, the species also seem to differ ecologically and occur in non-overlapping areas (see below).

Finally, another species that has often been associated with *Abutilonalbidum* is *A.fruticosum* Guill. & Perr. In fact, Maire (1933) reduced the latter to subspecies rank, as A.albidumsubsp.fruticosum (Guill. & Perr.) Maire (see also Quézel and Santa (1963)). A specimen of *A.albidum* at FI, annotated with ‘type’, is also stored under *A.fruticosum*. More or less similar criteria are also followed by POWO (2023) that considers *A.albidum* sensu Webb & Berthel. as a synonym of *A.fruticosum*. Hochreutiner (1902) already emphasised that these two species are, in fact, not related. *Abutilonfruticosum* has smaller and paler (green turning brown, never blackish), almost turbinate fruits that always have fewer mericarps (usually 9–10), smaller rounded leaves, often with almost entire margins and a stem indumentum that entirely consists of very short stellate hairs.

### ﻿The species of *Abutilon* relevant to this study can be identified using the following key

See also Figs 1–5.

**Table:** 

1	Perennial shrub; corolla large, 25–35 mm in diameter; calyx more or less urceolate, strongly keeled and cordate-saccate basally; schizocarp with ca. 10 mericarps, each mericarp with 3–5 seeds; naturalised garden escape	***A.grandifolium***
–	Annual herb or perennial (sub-)shrub; corolla often smaller, 10–35 mm across; calyx neither urceolate, keeled nor cordate at base; schizocarp with ca. 10 to numerous mericarps, each with 1–3 seeds; weedy and/or non-ornamental species	**2**
2	Annual herb with stem indumentum of almost exclusively short hairs; corolla orange yellow; mericarp with dorsal cusps up to 5 mm long	***A.theophrasti***
–	Perennial subshrub with stem indumentum of short stellate hairs, often also with either multicellular hairs or long simple hairs; corolla pale to orange yellow; mericarps with dorsal margin variable, more or less rounded, acute or cuspidate (cusps, if present, up to 2 mm long)	**3**
3	Flowers 25–35 mm in diameter, with staminal column 5–7 mm long; mericarps 15–22 in number, 10–18 × 7–9 mm	***A.indicum***
–	Flowers always smaller, 15–18 mm in diameter or less, with staminal column 2–3 mm long; mericarps up to 16 in number, (5–) 8–10 × 3–5 mm	**4**
4	Stem indumentum exclusively of short, dense stellate hairs; leaves small, with almost entire margins; fruit almost turbinate; mericarps 9–10, almost rounded dorsally	***A.fruticosum***
–	Stem indumentum of short stellate hairs, multicellular hairs and long simple patent hairs; leaves with irregularly crenate-toothed margins; fruit not turbinate; mericarps more numerous, usually ca. 15, dorsally acute-triangular or with two cusps up to 2 mm long	**5**
5	Mericarps with (apical) dorsal margin distinctly cuspidate, each cusp 1–2 mm long	***A.bidentatum***
–	Mericarps merely acute at (apical) dorsal margin, without cusps	***A.albidum***

### Taxonomic treatment

#### ﻿ Abutilon albidum

Taxon classificationPlantaeMalvalesMalvaceae

(Willd.) Sweet, Hort. Brit.: 54. 1826.

3EB212C3-90BE-51A3-89B9-FA47DF4B5A26

≡ Abutilonalbidum (Willd.) Webb & Berthel., Hist. Nat. Iles Canaries (Phytogr. Canar.) Tome troisième, Deuxième partie, Sectio 1: 39. 1836, isonym.
≡ Abutilonindicumvar.albidum (Willd.) Baker f., J. Bot. 31: 213. 1893.
≡ Sidaalbida Willd., Enum. Pl.: 722.1809, nom. cons. prop.
Non Abutilonalbidum Hooker & Arn., Bot. Beechey’s Voy. 278. 1841, nom. illegit.

##### Type.

Spain. Teneriffa, Barranco Santo, *Webb* (FI barcode FI006084), typ. cons. prop. (Verloove and Sennikov 2022).

##### Description.

Erect, short-lived perennial herb or shrub up to 60(–100) cm tall. Branches densely covered by minute stellate hairs, mixed with numerous long, simple spreading hairs (especially, but not exclusively on new growth) and sparser multicellular hairs. Leaves (median cauline) up to 13 cm long and 7 cm wide, broadly ovate to almost rotund, deeply cordate at base, acute to acuminate at apex, irregularly crenate-toothed to double-toothed, very shortly stellate-pubescent on both sides; petiole up to 6 cm long, slightly shorter than blade, very densely stellate hairy mixed with rather numerous short, glandular, capitate hairs and scattered weak, simple, spreading hairs; longest stipules to 9 mm long (mostly shorter), filiform. Flowers axillary, mostly solitary, but sometimes merging to more or less distinct panicles, pedicel 2–4 cm long, elongating in fruit up to 1–8 cm, articulate and geniculate ca. 5 mm below the apex. Calyx 5-lobed for ca. ½ its length, sepals up to 8 mm long, densely long-pubescent on both sides, slightly accrescent, erect, ultimately reflexed; lobes lanceolate-acuminate. Corolla pale yellow to yellow, ca. 1.5 cm across; petals 7–10 mm long, 4–5 mm wide, obovate. Staminal column 2–3 mm long, stellate-pubescent. Fruit 8–10 mm long, ± 13 mm across; mericarps 10–15, black and rather star-like spreading at maturity, up to 9 mm long, 5 mm wide, the outer apical (dorsal) angle acute-triangular, without protuberances, stellate-pubescent towards the margin. Seeds 2(–3), brown, initially with scattered stellate hairs, pilose near margins, ca. 2 mm across.

##### Illustrations.

**Figure 1.**
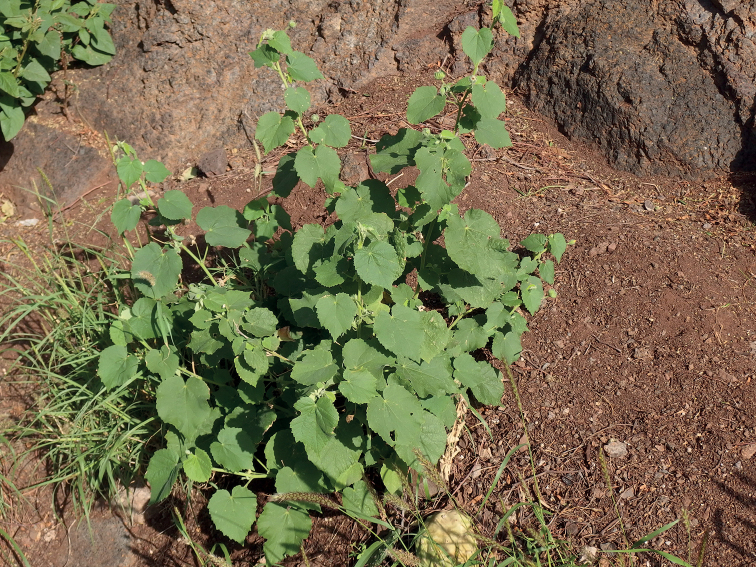
*Abutilonalbidum* in Igueste de San Andrés, Tenerife. General habit of plants growing below rocks along the side of the road. December 2019, F. Verloove.

**Figure 2.**
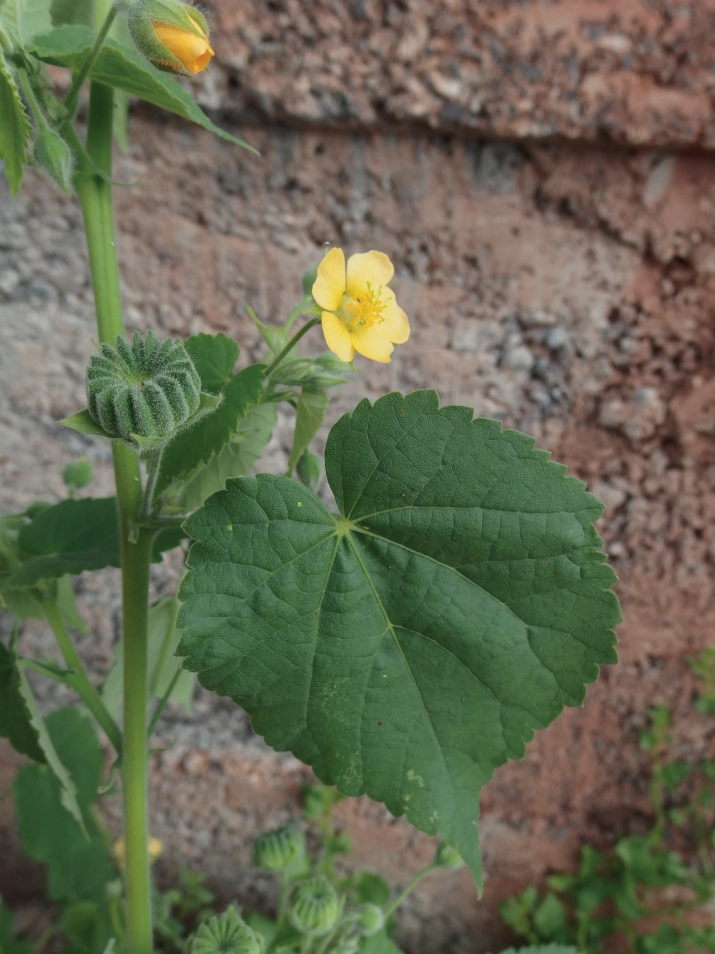
*Abutilonalbidum* in Igueste de San Andrés, Tenerife. Details of flower and immature fruit. December 2019, F. Verloove.

**Figure 3.**
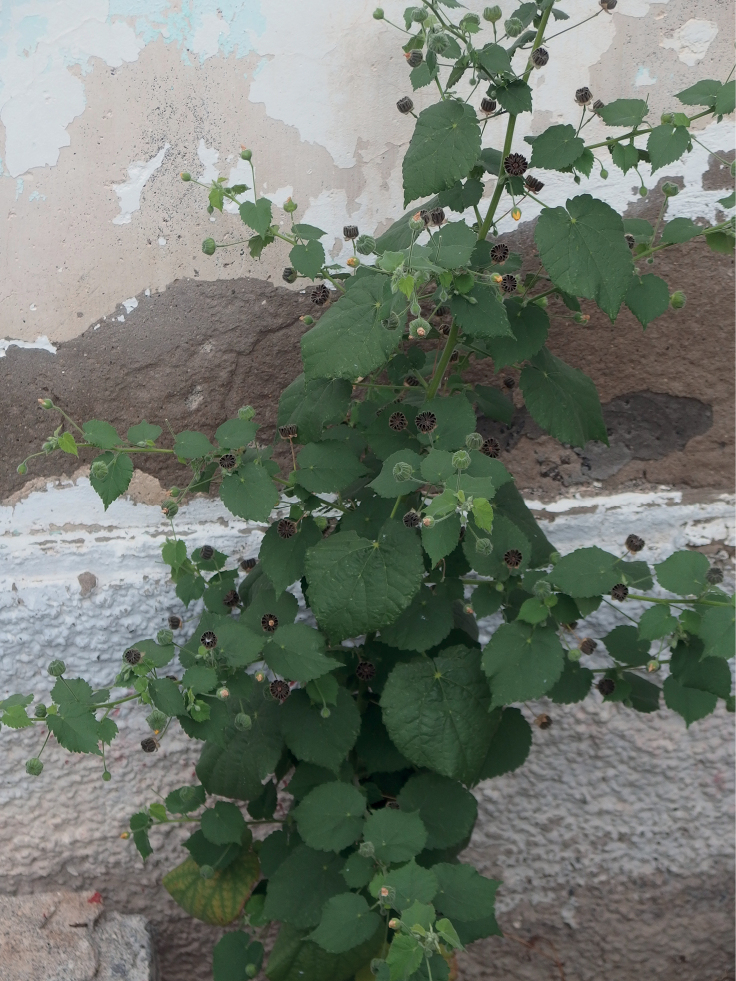
*Abutilonalbidum* in Igueste de San Andrés, Tenerife. Flowering and fruiting individual. December 2019, F. Verloove. In Tenerife, this species is now mostly found in anthropogenic habitats.

**Figure 4.**
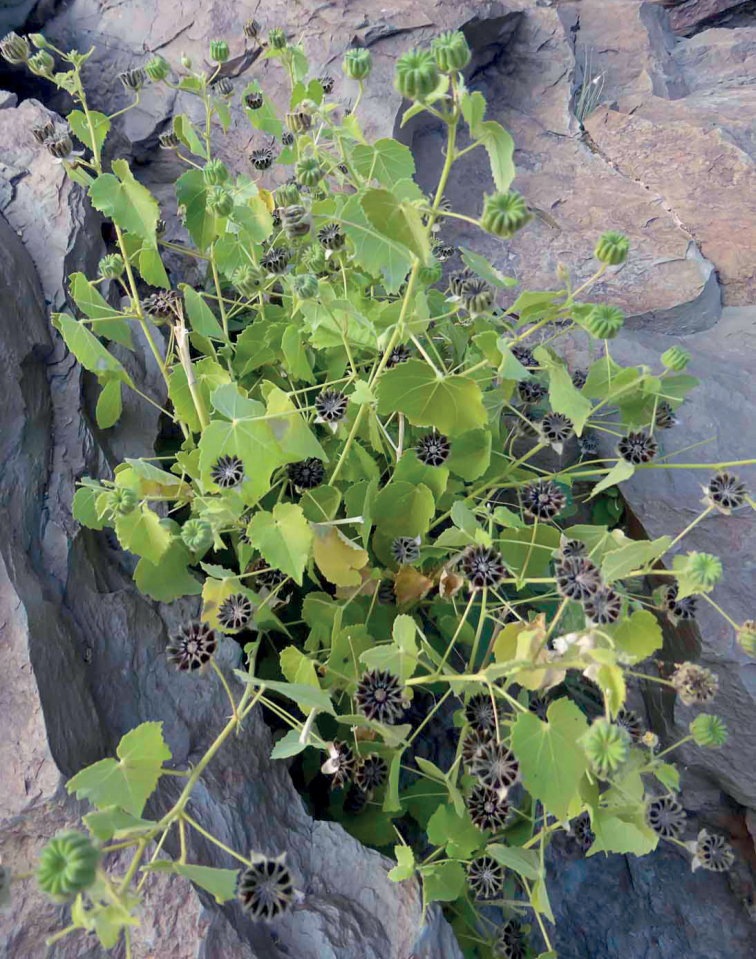
*Abutilonalbidum* near Tiglit, Morocco, December 2016, A. Garcin. In Morocco, this species is usually found in more natural habitats, often in crevices of basaltic rocks in (semi-) desert areas.

Webb and Berthelot (1836) presented the first superb line drawing of *Abutilonalbidum*. Figs 1–4 show the species in nature in the Canary Islands (Tenerife) and Morocco (Tiglit), respectively.

**Figure 5.**
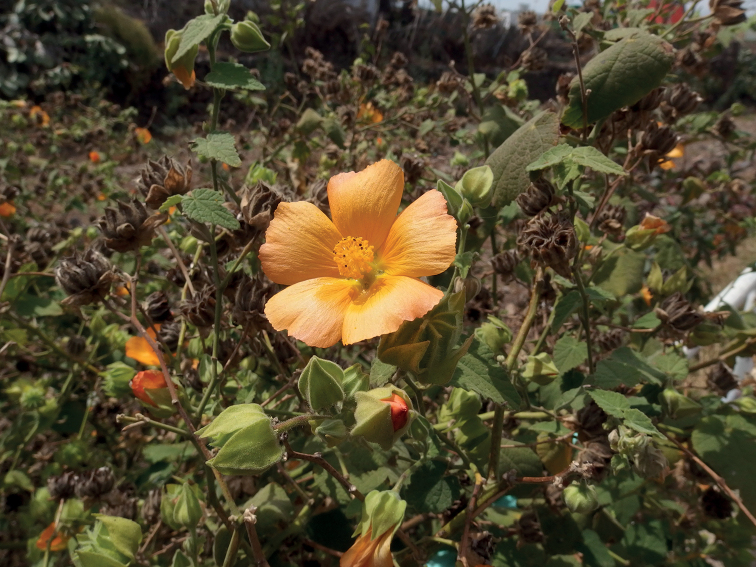
*Abutilongrandifolium* in Telde, Gran Canaria. April 2017, F. Verloove. For many decades, *A.albidum* was erroneously considered to be a synonym of this invasive species from South America. *Abutilongrandifolium* has much larger, more orange-yellow corollas and sepals forming longitudinal keels in bud and saccate at base.

##### Distribution.

**Figure 6.**
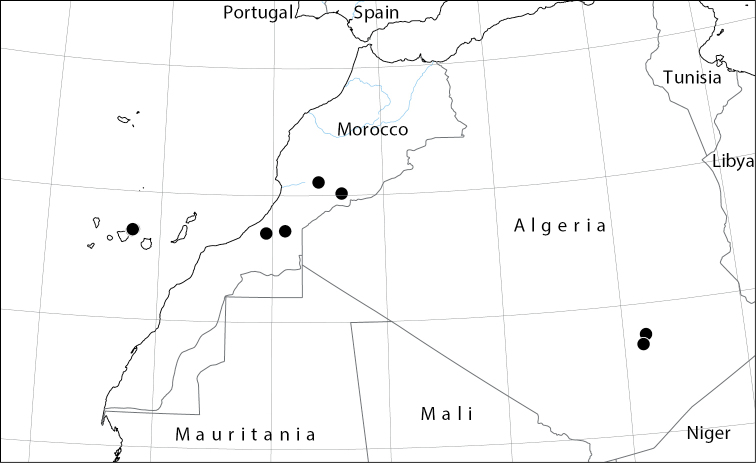
Worldwide distribution of *Abutilonalbidum* (historical and current records).

Macaronesia (Spain: Canary Islands), north-western Africa (Algeria: Hoggar Mountains; Morocco: Anti-Atlas) (Fig. 6).

**Figure 7.**
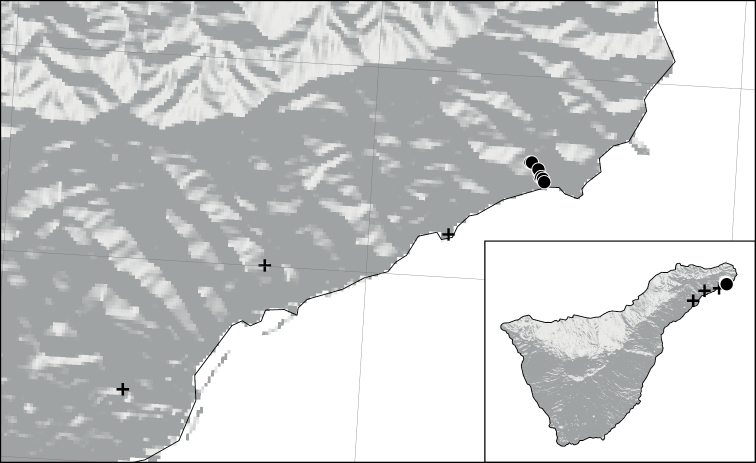
Old (black cross) and recent (black bullet) distribution of *Abutilonalbidum* in Tenerife, Canary Islands, Spain.

Webb and Berthelot (1836) reported *Abutilonalbidum* from ‘Barranco Santo’, near the Zurita Bridge, in Santa Cruz de Tenerife, in the south-eastern part of the Island. It had previously been collected from Tenerife in 1796 by A. de Jussieu (P!; see specimens examined). It was considered a Canarian endemic (Masferrer y Arquimbau 1880). Nearly all collections that have been made since then originated from the Barranco de Santos Ravine (present-day name) in Santa Cruz (Fig. 7). Historical literature sources mostly also referred to that locality (e.g. Masferrer y Arquimbau (1880); Bornmüller (1904)). Some further historical literature sources reported it from additional localities in Tenerife. According to Lindinger (1926), it was also observed in La Orotava, ca. 35 km further SW. Pitard and Proust (1908) also mentioned it from another ravine in Santa Cruz, the Barranco del Bufadero. The species was said to be very rare in Tenerife. Burchard (1929) probably presented the most extensive (and most recent) update of its occurrence on the Island. It was mostly seen between San Andrés and Igueste [F.V.: i.e. Igueste de San Andrés], including below the Los Órganos cliffs [F.V.: i.e. north of the Las Teresitas Beach]. It was also said to have isolated occurrences in Santa Cruz, where it had probably become extinct. All documented occurrences, except for a single record from La Orotava, were from the extreme south-eastern part of the Island where it occupied an area of hardly more than 12 km^2^. In Tenerife, it was probably last collected in 1945 by E.R. Sventenius. According to some sources, it was formerly also collected in Gran Canaria (Barranco de la Angostura) (Pitard and Proust 1908); unfortunately, no specimens are present in the LPA herbarium.

**Figure 8.**
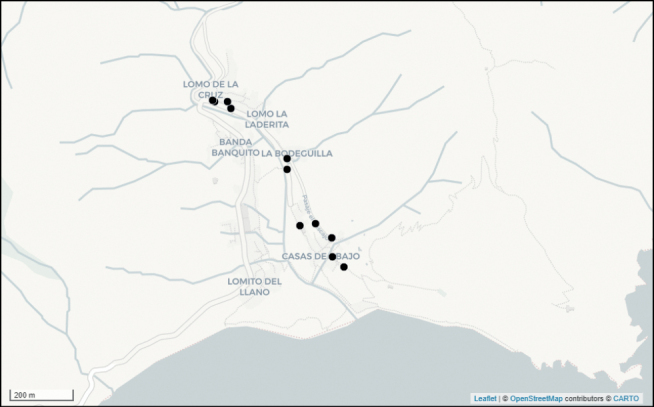
Distribution of recently discovered populations of *Abutilonalbidum* in Igueste de San Andrés in Tenerife, Canary Islands, Spain.

In December 2019, *Abutilonalbidum* was rediscovered in Tenerife in Igueste de San Andrés, 74 years after Sventenius’ collection from 1945. In the same village, several small populations of identical plants were observed in December 2019. Some plants were seen along the side of the road Carretera de Igueste de San Andrés adjacent to the Barranco de Igueste (these plants are also visible on Google Streetview images from June 2012: https://www.google.com/maps/@28.5291262,-16.1568142,3a,75y,15.61h,60.37t/data=!3m6!1e1!3m4!1s6-0-oKg77q5kAJSCf1_wCQ!2e0!7i13312!8i6656). Further plants were seen as weeds in a plantation near the San Pedro Apóstol church. Finally, the species was also observed on both sides of the Paseo el Cementerio, a track that leads to the cemetery. In March 2022, a more detailed survey was carried out (Fig. 8): the species was found in several additional localities, but less than 100 individuals in total and in an area of at most 35,000 m^2^ (for details see: https://observation.org/soort/view/1029740). Interestingly, in the very same area, the Belgian amateur botanist Leon Delvosalle (1915–2012) collected this species in May 1962, but erroneously identified it as *Sidarhombifolia*, a subtropical Malvaceae weed that is also naturalised there. After the Meise Botanic Garden (BR) acquired his herbarium, this collection (consisting of four small fragments only) was re-identified by the first author as Abutiloncf.indicum in March 2013. All these localities fall within the distribution area as described by Burchard (1929). From this, it can be deduced that *A.albidum* has survived at least in Igueste de San Andrés in Tenerife. Meanwhile, Santa Cruz de Tenerife became the capital of the Island and has dramatically changed in the past century. Repeated botanical explorations over the years in the ravines where *A.albidum* formerly occurred, especially Barranco de Santos and Barranco del Bufadero, were fruitless. Additional fieldworks in May and June 2021 in potentially suitable areas in Barranco Tahodio, Barranco Valleseco, between Igueste de San Andrés and San Andrés, Playa de las Teresitas (Bajo Los Órganos) and in Playa de Las Gaviotas (Bajo Los Órganos) were also unsuccessful. The coastal area between Santa Cruz and San Andrés was mostly lost due to the expansion of the commercial port and the urbanisation of the capital. Moreover, the highly invasive grass species *Cenchrussetaceus* (Forssk.) Morrone has now colonised the entire area, including the habitats where *A.albidum* once occurred. In Los Órganos, the species probably grew near the old path between Las Teresitas and Las Gaviotas that goes through the base of the basaltic rocks of Los Órganos.

After its discovery in Tenerife, *Abutilonalbidum* also was found in north-western Africa. Maire (1933) first reported it from Ideles and Imarera in the Hoggar Mountains, a highland region in the central Sahara in southern Algeria, along the Tropic of Cancer, where it was said to be rare (Quézel and Santa 1963). Its presence there has not been confirmed lately and it is likely extinct (pers. comm., Prof. Said Amrani, January 2022). From Morocco, it was first reported from Assa in the southwest of the country (Maire 1936; Emberger and Maire 1941). Later, it was also reported from Foum Zguid in south-eastern Morocco (Fennane et al. 1999) and Maire collected it in 1937 in “Tiliouine” (Taliouine), slightly further north (UM-MPU-MPU084348!). There are apparently no recent herbarium collections or more recent reports of the species; however, it is still present at least in the surroundings of Tiglit in Morocco (https://www.teline.fr/en/photos/malvaceae/abutilon-albidum), where it had been confused with the introduced weed *A.theophrasti*. *Abutilonalbidum* is probably rare throughout its entire distribution range and probably poorly known as well. It was assessed as endangered in a preliminary Red List of the Moroccon flora (Fennane 2018).

##### Habitat and ecology.

In Algeria and Morocco, *Abutilonalbidum* usually occurs on basaltic rocks in the desert or semi-desert, from subtropical to warm (Mediterranean) climate areas. It is most often found at higher elevations (up to 2,000 m above sea level), but may also grow at lower elevations. From herbarium labels, it can be deduced that the species also occurs in sandy, dried-out riverbeds. Habitats are usually natural and little disturbed although it was also collected (likely as a weed) in a palm grove (Maire 1936). It is nowhere in the world known as a weed (Randall 2017).

In Tenerife, *Abutilonalbidum* is best known from its *locus classicus*, i.e. the dried-out riverbed of Barranco de Santos in Santa Cruz. It was said to grow in dry rock crevices and warm ruderal places (Webb and Berthelot 1836; Bornmüller 1904; Pitard and Proust 1908). It formerly also was reported from basaltic rocks below the Los Órganos sea cliff (Burchard 1929). However, the most recent observations are almost exclusively from more degraded, anthropogenic habitats: roadsides, plantations and ruderal places, where it is found along with, for example, *Bituminariabituminosa* (L.) C.H. Stirt., *Cynodondactylon* (L.) Pers., *Fagoniacretica* L., *Forsskaoleaangustifolia* Retz., *Patellifolia* sp., *Setariaadhaerens* (Forssk.) Chiov. etc. In one locality, a few plants were also found on the basaltic rocks bordering the road; there, the species was accompanied by, amongst others, *Aristidaadscensionis* L., *Cenchrusciliaris* L. and *Kleinianeriifolia* Haw. In Tenerife, all populations, including the historical ones, are at elevations well below 100 m, often near to sea level.

##### Specimens examined.

**Spain. Canary Islands, Tenerife**: Teneriffa, Barranco Santo, *Webb* (FI006084, proposed conserved type); Canaries, s.d., herb. *Webb* (P06730933); Barranco Santo propè opp. Sta. Cruz, s.d., *Webb* (P06730936; BR); Ténérif, s.d., sine coll. (MNHN-P-P06731032); Barranco Sancto propè Sta. Cruz, s.d., sine coll. (BR 0000013462017); Teneriffe, 1796, *A. de Jussieu* (P06730938); Ténérife, Sa. Crux, 1816, *C. Smith* (G00219753, G00219752, G00219721); Teneriffa, in convalle aridiforme a Barranco Santo propè urbem Sanctam Cruceum, June 1834 (K000240407); Teneriffe, 1848, *S. Berthelot* (P06731022, K000240405, K000240406); Tenériffe, 1854, *C. Bolle* s.n. (COI00057130); Teneriffa, Barranco Santo près Santa Cruz, 12 April 1855, *E. Bourgeau* (P06730935, RAB078278); ibid. (P06641194); Barranco del Hierro, Sud-Est de Tenerife, 15 April 1855, *H. de la Perraudière* (P04694330); Reg. infer., Sud-Est de Tenerife, 15 April 1855, *H. de la Perraudière* (P06731031); Prope Sta. Crux, 15 April 1855, *H. de la Perraudière* (P06731023); ibid. (P06731030); ibid. (MPU748052); Barranco Santo, 1866, *T. Husnot* (Pl. Canarienses 632) (P04642214); ibid. (P04694328); ibid. (P06641193); ibid. (MPU748054); Teneriffa, Santa Cruz, Bco. Santo (loc. class.), 15 June 1901, *J. Bornmüller* 2132 (P06731034; BR; MPU748050); ibid. (P06731033); Inter Sanctum Andream et Igueste oppidula Teneriffae, in rup aridis, 3 May 1907, *O. Burchard* (ORT 00001); San Andrés, Los Órganos, sitios rocosos y secos, +/- escasa, 3 April 1944, *E.R. Sventenius* (ORT 12332); San Andrés, Roque de Los Órganos, escasa, 11 January 1945, *E.R. Sventenius* (ORT 12331); Igueste [de San Andrés], bord du chemin, 5 May 1962, *L. Delvosalle* 5235 (BR); Santa Cruz de Tenerife, Igueste de San Andrés, Carretera de Igueste de San Andrés N of the Barranco de Igueste, roadside, few plants, 23 December 2019, *F. Verloove* 13743 (BR); Santa Cruz de Tenerife, Igueste de San Andrés, Paseo el Cementerio, alongside track, on both sides, ca. 15–20 individuals, 23 December 2019, *F. Verloove* 13744 (BR); Santa Cruz de Tenerife, Igueste de San Andrés, Pasaje El Cascajo, roadside, scattered individuals, 26 March 2022, *F. Verloove* 14278 (BR).

## Supplementary Material

XML Treatment for Abutilon albidum
